# Supplementary material for: Cellulose Nanofibrils/Xyloglucan Bio-Based Aerogels with Shape Recovery
Source: Gels. 2021 Jan 5;7(1):5. doi: 10.3390/gels7010005 (PMC7839008; doi:10.3390/gels7010005)
Supplement: Supplementary file 1 [file gels-07-00005-s001.pdf]

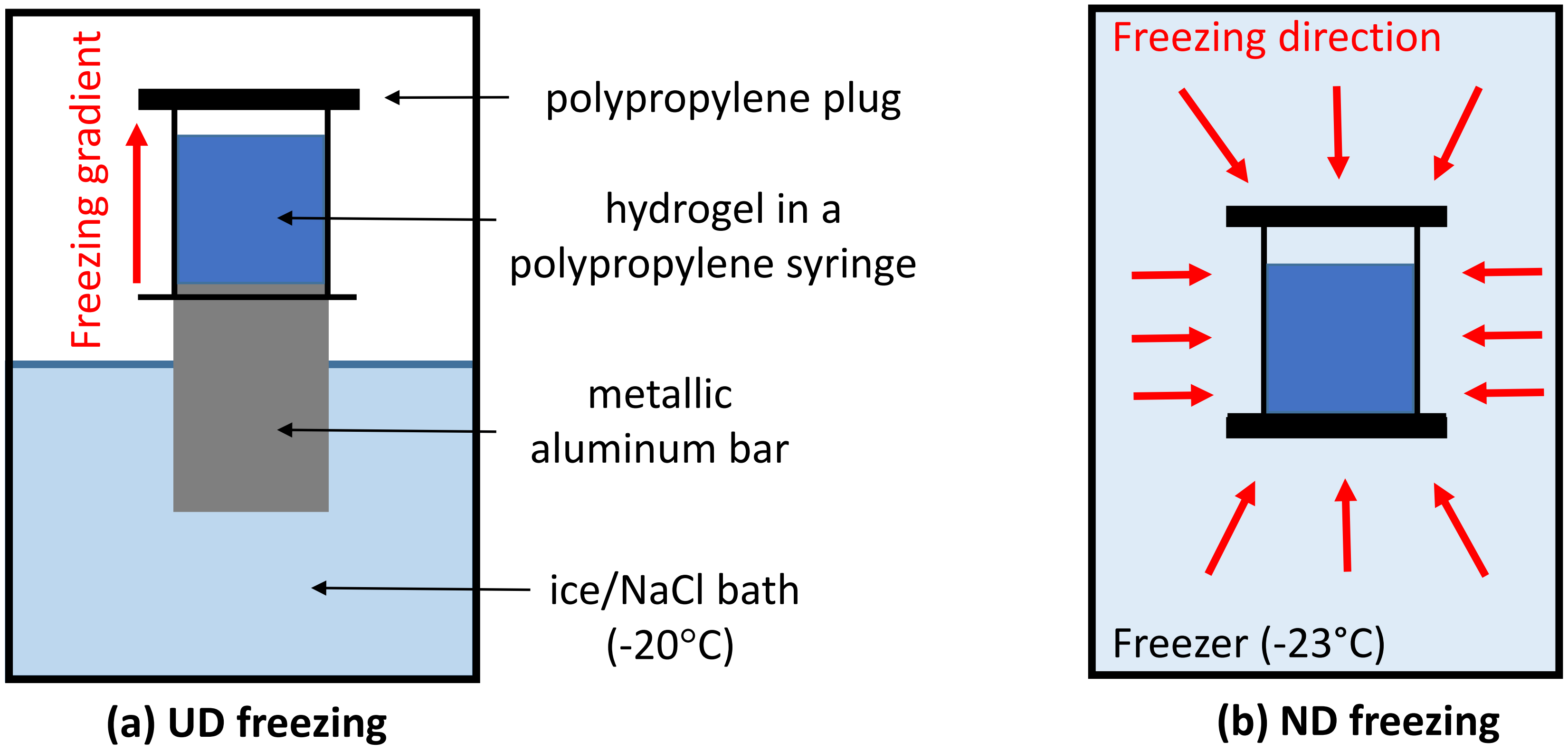

Figure S1. Schematic representation of the freezing conditions used in this work. (a) Unidirectional (UD) freezing condition. The polypropylene syringe containing the hydrogel is mounted on the top surface of a metallic aluminum bar which is placed vertically into ice/NaCl bath at -20°C to allow a vertical freezing gradient across the sample. (b) non-directional (ND) freezing, the polypropylene seringue containing the hydrogel is placed in a freezer at -23°C to allow isotropic freezing of the sample.
